# DomArchive: a century of published dominance data

**DOI:** 10.1098/rstb.2020.0436

**Published:** 2022-02-28

**Authors:** Eli D. Strauss, Alex R. DeCasien, Gabriela Galindo, Elizabeth A. Hobson, Daizaburo Shizuka, James P. Curley

**Affiliations:** ^1^ Department of Collective Behavior, Max Planck Institute of Animal Behavior, 78464 Konstanz, Germany; ^2^ Centre for the Advanced Study of Collective Behaviour, University of Konstanz, 78464 Konstanz, Germany; ^3^ School of Biological Sciences, University of Nebraska Lincoln, Lincoln, NE, 68588-0118 USA; ^4^ Department of Anthropology, New York University, New York, NY, USA; ^5^ New York Consortium in Evolutionary Primatology, New York, NY, USA; ^6^ Section on Developmental Neurogenomics, National Institute of Mental Health, Bethesda, MA, USA; ^7^ Department of Biological Sciences, University of Cincinnati, Cincinnati, OH, USA; ^8^ Department of Psychology, University of Texas at Austin, Austin, TX, USA

**Keywords:** dominance hierarchy, sociomatrix, agonism, aggression, submission, comparative biology

## Abstract

Dominance behaviours have been collected for many groups of animals since 1922 and serve as a foundation for research on social behaviour and social structure. Despite a wealth of data from the last century of research on dominance hierarchies, these data are only rarely used for comparative insight. Here, we aim to facilitate comparative studies of the structure and function of dominance hierarchies by compiling published dominance interaction datasets from the last 100 years of work. This compiled archive includes 436 datasets from 190 studies of 367 unique groups (mean group size 13.8, s.d. = 13.4) of 135 different species, totalling over 243 000 interactions. These data are presented in an R package alongside relevant metadata and a tool for subsetting the archive based on biological or methodological criteria. In this paper, we explain how to use the archive, discuss potential limitations of the data, and reflect on best practices in publishing dominance data based on our experience in assembling this dataset. This archive will serve as an important resource for future comparative studies and will promote the development of general unifying theories of dominance in behavioural ecology that can be grounded in testing with empirical data.

This article is part of the theme issue ‘The centennial of the pecking order: current state and future prospects for the study of dominance hierarchies’.

## 1. Introduction

Dominance is a pervasive feature of animal societies that can have dramatic effects on individual fitness. As a result, agonistic interactions—the individual aggressive and submissive signalling behaviours that underlie dominance hierarchies—are some of the most commonly collected behaviours across studies of animal [1–190]. These interactions are typically used to understand how within-group competition structures animal societies [191,192]. In most social species, individuals form dominance relationships, where agonistic interactions between any pair of individuals follow a predictable asymmetric pattern, where one member of the dyad typically yields to the other [193]. The dominance hierarchy is the group-level social structure that emerges from the network of dominance relationships, and various ranking methods have been developed to infer individual position in the dominance hierarchy based on the outcomes of observed agonistic interactions [194–196]. Individual position in the hierarchy is correlated with behaviour, physiology, gene expression, reproduction and longevity in many species (this issue: [197–205]). Higher-order patterns, such as the degree of linearity or transitivity of dominance relationships [206–208], or the amount of inequality in the outcomes of agonistic interactions [110] can reveal the overall structure of the dominance hierarchy in different societies [209]. Agonistic interactions sampled over time can be used to understand canonical patterns in sequences of interactions [210] or to infer the dynamics of social hierarchies [211–213].

Although agonistic interaction datasets are typically collected to address questions about the behaviour of a specific species, these datasets also have strong potential for comparative insight about the evolution of sociality in the face of competition. However, these data have only rarely been applied in a comparative framework to address evolutionary questions about competition and hierarchy structure (but see [206,207,214–217]).

Here, we aim to facilitate comparative study into dominance interactions and emergent aspects of hierarchical structure by assembling a comprehensive database of published agonistic interactions dating back to the first published ‘peck-orders’ in Schjelderup-Ebbe's research into dominance among domestic hens in 1922 [39]. The data are presented in an R package alongside metadata and tools for filtering the archive by its associated metadata (see electronic supplementary material for an instructional vignette).

## 2. The dominance archive dataset

**Figure 1.**
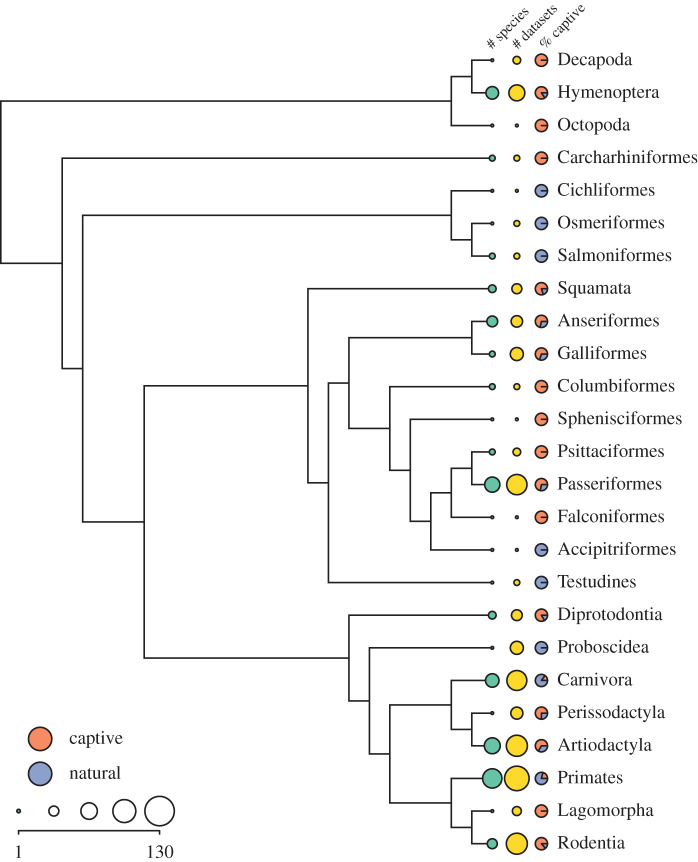
A phylogeny of taxonomic orders included in the archive, with counts of unique species and datasets in each order (dot sizes are log_10_ scaled, legend shows the corresponding untransformed sample sizes), and the percentage of datasets from captive versus wild populations. Phylogeny is from the Open Tree of Life [218]. Data for one order (Perciformes: 2 species, 3 datasets) are not included here due to paraphyly. (Online version in colour.)

The archive contains 436 agonistic interaction datasets from 190 studies [1–190] of 135 unique species (figure 1), totalling over 243 000 interactions. Because some animal social groups were sampled multiple times within a single study or over multiple studies, the archive includes data from 365 unique social groups (mean group size = 13.8, s.d. = 13.4). The last century has seen notable shifts in the ways researchers approach the study of dominance (Hobson [219]), the analytical approaches to measuring dominance [110,195,206,220], and the customs governing data storage and sharing [221]. This variation is reflected in the archive and is captured by metadata and summary statistics associated with each dataset (table 1; electronic supplementary material, data S1).

**Table 1.** Metadata and summary statistics associated with each dataset in the archive.

metadata column	meaning	potential values
fileid	unique identifier for data	
order	order (taxonomic rank)	
species	species name	
common_name	common name	
study_site	nation where study was conducted	
captivity	captive or free-ranging animals?	‘captive’, ‘natural’
sex	males, females, or both?	‘M’, ‘F’, ‘MF’
age	what age classes?	‘adult’, ‘non-adult’, ‘mixed’
measure	what behaviour was measured?	
data_location	where is data in reference (e.g. Tb1)?	
countbinary	are data raw counts or binary? (edgelists are counts)	‘count’, ‘binary’
repeat_group	are there multiple datasets for this group?	‘yes’, ‘no’
groupid	unique identifier for social group	
matrix_edgelist	are data in matrix or edgelist format?	‘matrix’, ‘edgelist’
edgelist_time_meaning	for edgelist data, what is the meaning (units) of the ‘time’ column	
note	miscellaneous notes	
full_citation	source for the data	
number_individuals	number of individuals in the dataset	
number_interactions	number of interactions in the dataset	
interactions_per_individual	number of interactions per individual	
proportion_unknown	proportion of relationships for which there are no observations (matrix data only)	

Most (*n* = 418) datasets are in the form of a sociomatrix, a square matrix documenting the outcomes of agonistic interactions over the study period (figure 1). During the last century, sociomatrix notation became the standard for presenting data on dominance interactions. In these matrices, the identities of winners of interactions—the individuals who elicit submission or avoidance in their opponents—are listed in the rows, and losers of interactions—exhibitors of submission or avoidance—are listed in the columns. The entries in the matrix correspond to the numbers of times the row individual was observed to dominate the column individual. In rare cases, these data were published in binary format, such that cells in the matrix are either 1 if the row individual was observed to dominate the column individual more often than vice versa and 0 otherwise. These ‘binary’ matrices are noted in the metadata (table 1). In all cases, the diagonal of the matrix is ‘NA’ because individuals cannot dominate themselves. Because sociomatrices tabulate interactions over the duration of some observation period, these datasets contain no information about the order in which interactions occurred.

Some (*n* = 18) datasets in the archive are in edgelist format, which presents dominance interactions listing the winner, the loser, the sequence in which the interactions occurred, and (in some cases) information on the timing of the interactions. This data format has become increasingly common in the last decade as ranking methods that incorporate sequence information (e.g. [220,222]) are becoming more popular, and as raw data is increasingly supplied in digital supplements rather than appearing directly in print. Because of this extra temporal information, these datasets are crucial for addressing questions about the dynamics of dominance [223], which occur over both short [224] and long [57] timescales.

In addition to these data-format metadata, the archive also includes biological and methodological metadata about the study. These metadata include demographic information about the animal social group (age-class and sex composition), taxonomic information about the study organism (species, order), behavioural information about the agonistic interaction (interaction type), whether the study was conducted in captivity or on wild animals, the country in which the study was conducted, and whether the group was sampled repeatedly. For age-class and sex composition, it is important to note that these often reflect the study design rather than the biology of the organism—for instance, many datasets were collected from mixed-age social groups, but only data on interactions among adults were recorded. Groups are denoted as mixed-sex if at least one male and one female was included in the dataset. Groups were considered ‘captive’ if they were housed in an enclosure for any part of the collection of dominance interaction data; this therefore includes zoo and laboratory studies as well as studies where wild animals were captured and temporarily observed in an enclosure. Interaction type describes the specific agonistic behaviours (e.g. threats, chasing, displacement, submission) that are represented in the dataset, as laid out by the original authors. Finally, social groups were considered ‘repeated’ if the same set of individuals were observed multiple times in close succession or if multiple behaviours and corresponding datasets were collected from the same set of individuals. Importantly, groups sampled over longer time-frames during which demographic processes occur (e.g. long-term observational studies) and groups where membership was fluid (and thus some individuals appear in multiple groups) were not considered ‘repeat’ groups.

Finally, the archive includes dataset summary statistics alongside these metadata. The number of individuals, number of interactions, and proportion of unknown relationships describe the sampling coverage of each dataset. Additionally, the archive includes calculated measures of the structure of dominance relationships for each dataset (table 2): directional consistency [145], triangle transitivity [206], linearity [226] and steepness [110]. These summary statistics are useful for comparative insight into the ecological and evolutionary determinants of hierarchy structure [206,207,217,227,228].

**Table 2.** Hierarchy structure measures associated with each matrix dataset in the archive.

measure (column name in metadata)	range	description	dataset criteria	source
directional consistency index (dci)	0–1	the average directional asymmetry in wins across dyads. 1 = all dyads have one individual who wins every interaction, 0 = all dyads are ties.	matrix_edgelist = ‘matrix’ countbinary = count	[145]
triangle transitivity index (ttri)	mostly 0–1, rarely <0	the proportion of triads in the network of dominance relationships that are transitive, scaled so that 0 = expected triangle transitivity under random interactions and 1 = perfectly transitive. Rarely, negative values can occur if dominance relationships are less transitive than expected under random interactions.	matrix_edgelist = ‘matrix’	[206]
modified Landau's h’ measure of linearity (modified_landaus_h)	0–1	a measure of the linearity of dominance relationships, or the degree to which dominance relationships show transitive properties. 0 = completely cyclical hierarchy, 1 = completely linear hierarchy. This value is biased downward with increasing proportions of unknown relationships [225]; triangle transitivity is recommended as an alternative measure [206].	matrix_edgelist = ‘matrix’ countbinary = count	[226]
hierarchy steepness (ds_steepness)	0–1	a measure of the differentiation in winning ability among individuals, calculated as the absolute value of the slope of a line fitted through the normalized David's Scores of all contestants. David's Scores measure an individual's winning tendency.	matrix_edgelist = ‘matrix’countbinary = count	[110]
		0 = all individuals have the same score, 1 = all individuals are maximally differentiated in their scores. This value is biased downward with increasing proportions of unknown relationships [225].		

## 3. Dataset assembly

The following search criteria were used to identify potential datasets for the archive. First, we searched Google Scholar and PubMed for any papers, book chapters or theses that: (1) had cited key papers used to measure various hierarchy metrics [110,195,196,206,220,226,229,230]; (2) had used software to calculate hierarchy matrices including all versions of SOCPROG [231], MatMan [232] and the compete [233] and aniDom R packages [234]; or (3) had included the keyword phrases ‘linear dominance’ and/or ‘social hierarchy’ but had not cited the above papers or software. We also identified older papers (pre 1983) by opportunistically examining the references of already identified papers. Finally, we included data from two previous papers that had collated several sociomatrices [206,214].

Individual papers, book chapters and theses and any supplementary information or data repositories associated with papers were then searched for the presence of a sociomatrix, edgelist or some other data format (e.g. pecking order) that could be converted to a sociomatrix. To be included in the archive, we applied the following inclusion criteria: (1) We only included datasets that contained interactions among individuals, so datasets reporting on agonistic interactions among groups or species were not included (e.g. [235,236]). (2) The group needed to contain at least six individuals, because this is the minimum number of individuals for calculating some measures of hierarchy structure [226]. (3) All individuals in the study had to be free to interact with any other member of the group—that is, this archive does not include ‘tournament’ style studies where individuals are repeatedly paired for dyadic competition where the outcomes are treated as reflecting an underlying hierarchy linking all individuals. These studies were excluded from the dataset because evidence from the latter half of this century of research suggests that social context (e.g. bystander effects, winner-loser effects) is a fundamental feature of dominance hierarchies [126,237,238]. (4) We excluded matrices where physiological manipulations had been used to examine their effects on the hierarchy structure (e.g. [116]).

## 4. Using the package

Users can install the latest version of the DomArchive R package using the command ‘devtools::install_github(‘DomArchive/DomArchive’, build_vignettes = TRUE)’ (installation requires the devtools package). The datasets are accompanied by ‘subset_archive()’, a flexible function for selecting data from the archive based on the metadata. This function accepts either a list of dataset identifiers, or subsetting can be achieved by providing a list of metadata column names and a list of values corresponding to those columns (see electronic supplementary material, Supplemental Data for plain-text copy of the metadata). A simple vignette accompanying the R package provides a tutorial for how to do this. The vignette (electronic supplementary material) can be accessed after installing the package by running ‘vignette(‘introduction’, package = ‘DomArchive’)’. Users wishing to report issues, suggest additions to the archive, or inquire about data sources can contact the authors or submit an Issue at https://github.com/DomArchive/DomArchive.

Users of the dominance archive should be aware of some limitations to these data. First of all, most data are in sociomatrix format, which does not capture the order in which interaction occurred, making these data not suitable for analyses that require interaction order (e.g. Elo-rating). For data sources that are in edgelist format, information on the order of interactions is preserved, but the timings of the interactions are still uncertain. Two adjacent observations could have occurred immediately one after the other, or could have been days or weeks apart. We include time data when available, but the temporal resolution of this data is variable among studies. Another limitation of note for these data is that the datasets varied considerably in the timespan over which the data were collected and the frequency of observation during the study. For instance, some data were collected during uninterrupted observation within a single day (e.g. [121]), whereas other datasets were collected over multiple years of non-continuous observation (e.g. [43]). When group membership was fluid (e.g. [121]) or when multiple studies focused on a social group over long periods with demographic turnover (e.g. [51,143]), groups with different group ids in the archive contain overlapping individuals. Finally, users of the data for comparative analyses should exercise caution when only one or a few datasets are available for a given species. In assembling these data, we found that authors often included only a subset of their total data in the manuscript (e.g. an example matrix from one of many study groups). The decision process for selecting which example dataset to include was not always evident from the paper, but sometimes authors would publish a particular example for some notable characteristics of that data. For instance, in a study of 31 flocks of willow tits, over 90% of flocks were found to have linear hierarchies, but the examples included in the publication were the flocks with nonlinear hierarchies, because those exceptions were the focus of the study [163]. In such cases, we included this information in the ‘note’ column of the archive, but it was often the case that no reason was given for which example data were shared in the publication. In comparative studies, users of the data should inspect the original sources of datasets for species with limited available data to ensure that characteristics inferred for that species reflect the typical behaviour and are not biased by the research focus of the original source.

## 5. Recommendations for publishing future dominance data

In collecting data for this archive, we noticed a culture shift in the way dominance data are used and published that merits discussion. In the twentieth century, it was common to publish sociomatrices of (at least some of) the interaction data used in dominance analyses (e.g. [86]). However, more recently, the practice of publishing the raw dominance interaction data in the manuscript has become less frequent, with much of this information either not appearing in the paper at all or appearing only in electronic supplementary material. The movement of this data to online supplements has the potential to greatly improve data availability because of the relaxation of constraints imposed by journal page limits, but it has been accompanied by new emerging challenges that stifle this potential. In particular, there has been a troubling trend towards sharing processed data rather than raw interaction data. In many papers, data that accompany the paper include calculated ranks or ratings associated with individuals in the study, but the raw interactions used to infer those ratings are omitted. Recent steps towards reproducibility and open science have emphasized publishing the analysis code and raw data to reproduce all steps of the analysis [221,239]—here, we echo this call, and highlight that for analyses including rank as a covariate, this entails sharing the raw interaction data used to infer those ranks. Finally, the increasing use of the Elo-rating method has led to a shift away from sociomatrices and towards data structures that include information about the sequence of interactions. This change has led to exciting new research into dominance and its dynamics [220,223], but has also led to new challenges for data sharing. Whereas sociomatrices are standardized data structures, the edgelist datasets we assembled were much more variable in their structure, and the metadata associated with the data were often incomplete or difficult to interpret. These issues are likely in part driven by the reduced scrutiny during peer review paid to data and electronic supplementary material compared to the sociomatrices that used to appear in the main text of the paper. To facilitate data sharing and comparative research, we recommend that researchers publishing edgelist data include columns for the group identifier, the sequence number of the interaction, the identity of the winner, the identity of the loser, and a date or time column giving as precise a measure of the timing of the interaction as possible.

## 6. Conclusion

Dominance interaction data are widely collected and used to gain insight into the structure of animal societies. Here, we compile previously published data to encourage comparative insight into animal social hierarchies—insight which has been surprisingly sparse despite the potential of existing data. We look forward to building on this insight and expanding this archive in the years to come.

## Data accessibility

Data presented in this paper are available in the DomArchive R package at https://github.com/DomArchive/DomArchive.
